# Synopsis of *Nekemias* Raf., a segregate genus from *Ampelopsis* Michx. (Vitaceae) disjunct between eastern/southeastern Asia and eastern North America, with ten new combinations

**DOI:** 10.3897/phytokeys.42.7704

**Published:** 2014-09-16

**Authors:** Jun Wen, John Boggan, Ze-Long Nie

**Affiliations:** 1 Department of Botany, MRC-166 Smithsonian Institution, P.O. Box 37012, Washington D.C. 20013-7012, USA; 2 Key Laboratory for Plant Diversity and Biogeography of East Asia, Kunming Institute of Botany, Chinese Academy of Sciences, Kunming 650201, Yunnan, China; 3 Key Laboratory of Plant Resources Conservation and Utilization and College of Biology and Environmental Sciences, Jishou University, Jishou, 416000, Hunan, China

**Keywords:** *Ampelopsis*, Asia, eastern North America, *Nekemias*, Vitaceae

## Abstract

The genus *Nekemias* (Vitaceae) was first recognized by Rafinesque in 1838. It has been treated as a synonym of *Ampelopsis* Michx. Recent phylogenetic studies suggest that *Ampelopsis* as traditionally delimited is paraphyletic. To maintain the monophyly of each of the genera of Vitaceae, we herein segregate the Ampelopsis sect. Leeaceifoliae lineage from *Ampelopsis* and recognize these taxa in *Nekemias* Raf., which has a disjunct distribution in eastern to southeastern Asia and eastern North America. Nomenclatural changes are made for nine species and one variety: *Nekemias arborea* (L.) J. Wen & Boggan, *Nekemias cantoniensis* (Hook. & Arn.) J. Wen & Z.L. Nie, *Nekemias celebica* (Suess.) J. Wen & Boggan, *Nekemias chaffanjonii* (H. Lév. & Van.) J. Wen & Z.L. Nie, *Nekemias gongshanensis* (C.L. Li) J. Wen & Z.L. Nie, *Nekemias grossedentata* (Hand.-Mazz.) J. Wen & Z.L. Nie, *Nekemias hypoglauca* (Hance) J. Wen & Z.L. Nie, *Nekemias megalophylla* (Diels & Gilg) J. Wen & Z.L. Nie, Nekemias megalophylla var. jiangxiensis (W.T. Wang) J. Wen & Z.L. Nie, and *Nekemias rubifolia* (Wall.) J. Wen & Z.L. Nie. A taxonomic key is provided for the genus to facilitate identification.

## Introduction

*Ampelopsis* Michx. (Vitaceae) is one of the 15 recognized genera in Vitaceae with about 25 species (Suessenguth 1953; Chen et al. 2007; Wen 2007; Wen et al. 2013a). The genus was shown to be paraphyletic by recent phylogenetic analyses based on plastid (Soejima and Wen 2006; Ren et al. 2011; Nie et al. 2012) and nuclear *GAI1* (Wen et al. 2007) analyses. The plastid data (Soejima and Wen 2006; Ren et al. 2011; Nie et al. 2012) supported that the African *Rhoicissus* and the South American *Cissus striata* complex formed a clade with the simple or palmately leaved *Ampelopsis* (sect. *Ampelopsis*), while the nuclear data (Wen et al. 2007) suggested that they were more closely related to the pinnately leaved *Ampelopsis* (sect. *Leeaceifoliae*, as designated by Galet 1967 in the unpublished thesis). All analyses so far have supported the monophyly of each of the two sections based on leaf morphology (Galet 1967). Apart from the differences in leaf morphology, the two sections also differ in their axillary buds, with taxa in sect. *Ampelopsis* having serial accessory buds, and those in sect. *Leeaceifoliae* having complex axillary buds as in *Vitis vinifera* (Bernard 1972–1973; Gerrath and Posluszny 1989; Soejima and Wen 2006; Wen et al. 2007).

Within Vitaceae, five or six main clades are well supported based on analysis of molecular sequence data with the *Ampelopsis*-*Rhoicissus*-*Cissus striata* clade as one of these major clades of Vitaceae (Soejima and Wen 2006; Wen et al. 2007, 2013b; Ren et al. 2011; Liu et al. 2013). Ingrouille et al. (2002), however, resolved *Ampelopsis* as the basalmost branch of Vitaceae albeit with no support, and further argued that the presence of pinnate leaves, the thick corolla, and the floral and vegetative development in *Ampelopsis* were the least-derived characters within Vitaceae as compared with those in the outgroup taxa from Leeaceae. To maintain the monophyly of each of the genera of Vitaceae, we herein segregate the Ampelopsis sect. Leeaceifoliae lineage from *Ampelopsis*. Rafinesque (1838) established the genus *Nekemias* with *Ampelopsis bipinnata* Michx. [= *Ampelopsis arborea* (L.) Koehne] possessing pinnately compound leaves as the type species. *Nekemias* has been rarely mentioned in subsequent taxonomic work on Vitaceae or treated as a synonym of *Ampelopsis* (Merrill 1949; Suessenguth 1953; Wen 2007), but it represents the earliest name at the generic rank for the pinnately-compound leaved *Ampelopsis* clade.

## Taxonomic synopsis

### Nekemias

Taxon classificationAnimaliaVitalesVitaceae

Raf., Sylva Tellur. 87. 1838.

Ampelopsis Michx., pro parte

#### Woody climbers.

Branchlets with prominent lenticels. Pith white, continuous through nodes. Tendrils leaf-opposed, mostly bifurcate to sometimes trifurcate, and lacking adhesive discs. Leaves alternate, petiolate, stipulate, pinnately to ternately bipinnately or sometimes tripinnately compound. Inflorescences bifurcately compound cymes, long peduncled, leaf-opposite. Flowers pedicellate, mostly bisexual; calyx saucer-like; corolla of 5 thick petals; stamens 5, opposite to petals; disc adnate to the base of the ovary; ovary 2-locular, style short, conical, stigma rounded. Fruit a berry, globose or subglobose, purple, blue or black, 1-4 seeded. Seeds obovoid.

#### Type species.

*Nekemias bipinnata* (Michx.) Raf. [= *Nekemias arborea* (L.) J. Wen & Boggan].

Nine species with eight occurring in warm temperate to tropical areas of eastern and southeastern Asia (Suessenguth 1940; Galet 1967; Chen et al. 2007), and one species distributed in eastern North America extending to the Caribbean (Brizicky 1965). This intercontinental disjunct distribution between eastern Asia and eastern North America represent a classical biogeographic pattern of the Northern Hemisphere (Wen 1999; Wen et al. 2010).

Below we provide a taxonomic synopsis for the genus.

### 1. Nekemias arborea

Taxon classificationAnimaliaVitalesVitaceae

(L.) J. Wen & Boggan comb. nov.

urn:lsid:ipni.org:names:77142315-1

Figure 1A–B

Vitis arborea L., Sp. Pl. 1: 203. 1753. Basionym
Ampelopsis bipinnata Michx., Fl. Bor.-Amer. 1: 160. 1803, nom. illeg.
Cissus stans Pers., Syn. Pl. 1: 143. 1805, nom. illeg.
Cissus bipinnata (Michx.) Nutt., Gen. N. Amer. Pl. 1: 144. 1818, nom. illeg.
Nekemias bipinnata (Michx.) Raf., Sylva Tellur. 87. 1838, nom. illeg.
Vitis bipinnata (Michx.) Torrey & A. Gray, Fl. N. Amer. 1: 243. 1838, nom. illeg.
Cissus arborea (L.) Des Moulins in Durand, Actes Soc. Linn. Bordeaux 24: 156. 1862.
Ampelopsis arborea (L.) Koehne, Deutsch. Dendrol. 400. 1893.
Ampelopsis arborea (L.) Rusby, Mem. Torrey Bot. Club 5: 221. 1894, comb. superfl.

#### Distribution.

USA (Alabama, Arkansas, Florida, Georgia, Illinois, Indiana, Kentucky, Louisiana, Mississippi, Missouri, New Mexico, North Carolina, Ohio, Oklahoma, South Carolina, Tennessee, Texas, Virginia and West Virginia) and the Caribbean.

**Figure 1.**
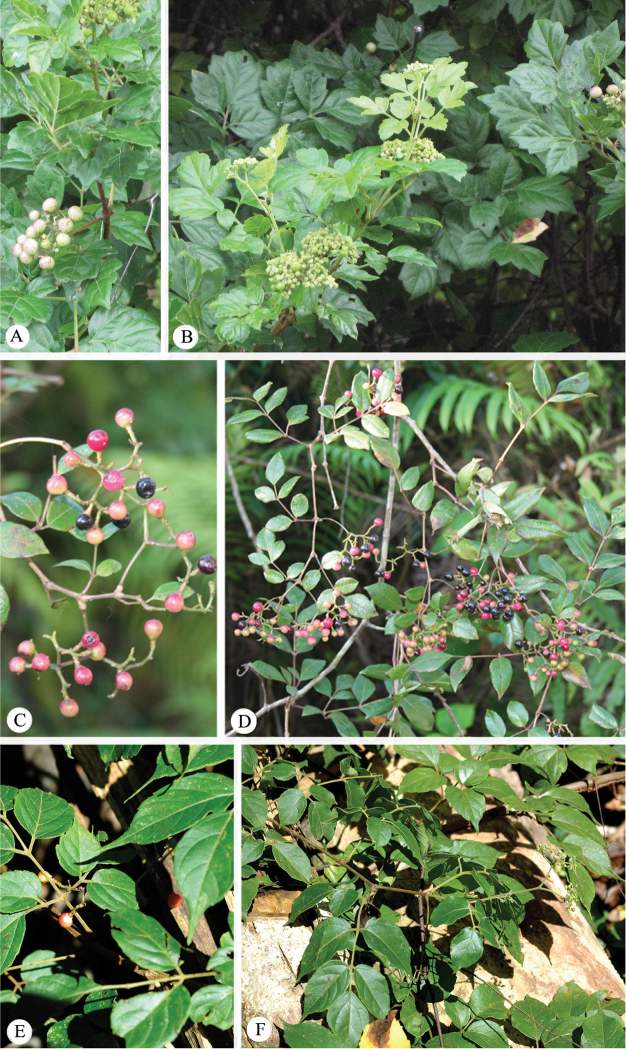
Images of representative species of *Nekemias* Raf. **A–B** *Nekemias arborea* (L.) J. Wen & Boggan, voucher specimen: *J. Wen 12005* (US), collected from Montgomery Co., Texas, USA **C–D** *Nekemias cantoniensis* (Hook. & Arn.) J. Wen & Z.L. Nie, voucher specimen: *J. Wen 10613* (US), collected from Xichou Xian, Yunnan province, China **E–F** *Nekemias celebica* (Suess.) J. Wen & Boggan, voucher specimen: *J. Wen 10242* (US), collected from SE Sulawesi, Indonesia.

### 2. Nekemias cantoniensis

Taxon classificationAnimaliaVitalesVitaceae

(Hook. & Arn.) J. Wen & Z.L. Nie comb. nov.

urn:lsid:ipni.org:names:77142316-1

Figure 1C–D

Cissus cantoniensis Hook. & Arn., Bot. Beech. Voy.: 175. 1833. Basionym
Cissus diversifolia Walp., Nov. Actorum Acad. Caes. Leop.-Carol. Nat. Cur. 19 (Suppl. 1): 314. 1843, nom. illeg., non DC. 1824.
Ampelopsis cantoniensis (Hook. & Arn.) K. Koch, Hort. Dendrol. 48: 11. 1853.
Vitis leeoides Maxim., Mel. Biol. Acad. Sci. St. Petersb. 9: 148. 1873.
Vitis cantoniensis (Hook. & Arn.) Seem., Bot. Voy. Herald: 370. 1875.
Ampelopsis cantoniensis (Hook. & Arn.) Planch., Monogr. Phan. 5: 460. 1887, comb. superfl.
Ampelopsis cantoniensis var. harmandii Planch., Monogr. Phan. 5: 460. 1887.
Ampelopsis leeoides (Maxim.) Planch. [epithet published in error as “*lecoides*” by Planchon in 1887], Monogr. Phan. 5: 462. 1887.
Ampelopsis loureiroi Hort. Mazel. ex Planch., Monogr. Phan. 5: 461. 1887, nom. nud. pro syn.
Vitis multijugata H. Lév. & Vaniot, Bull. Soc. Agric. Sci. Arts Sarthe 40: 41. 1905.
Leea theifera H. Lév., Repert. Spec. Nov. Regni Veg. 8: 58. 1910.
Ampelopsis annamensis Gagnep., Bull. Soc. Bot. France 92: 166. 1946.
Ampelopsis cantoniensis var. leeoides (Maxim.) F.Y. Lu [published in error as “*leecoides*”; changed to “*lecoides*” in Index Kewensis], Fl. Taiwan 3: 667. 1977, nom. invalid.

#### Distribution.

China (Anhui, Fujian, Guangdong, Guangxi, Guizhou, Hainan, Hong Kong, Hubei, Hunan, Taiwan, Xizang, Yunnan and Zhejiang), India, Japan (including the Ryukyu Islands), Vietnam, Laos, Malaysia (peninsular), and Indonesia (Java).

### 3. Nekemias celebica

Taxon classificationAnimaliaVitalesVitaceae

(Suess.) J. Wen & Boggan comb. nov.

urn:lsid:ipni.org:names:77142317-1

Figure 1E–F

Ampelopsis celebica Suess., Repert. Spec. Nov. Regni Veg. 49: 14. 1940. Basionym

#### Distribution.

Indonesia (Sulawesi).

### 4. Nekemias chaffanjonii

Taxon classificationAnimaliaVitalesVitaceae

(H. Lév. & Van.) J. Wen & Z.L. Nie comb. nov.

urn:lsid:ipni.org:names:77142323-1

Vitis chaffanjonii H. Lév. & Van., Bull. Soc. Agric. Sci. Arts Sarthe 40: 37. 1905. Basionym
Leea dielsii H. Lév., Repert. Spec. Nov. Regni Veg. 8: 58. 1910.
Meliosma cavaleriei H. Lév., Repert. Spec. Nov. Regni Veg. 9: 457. 1911.
Ampelopsis watsoniana E.H. Wilson, J. Roy. Hort. Soc. 42: 37. 1916, nom. nud.
Vitis watsoniana (E.H. Wilson) Bean, Trees & Shrubs Brit. Isles 2: 673. 1921.
Ampelopsis chaffanjonii (H. Lév. & Van.) Rehder, J. Arnold Arbor. 15: 25. 1934.

#### Distribution.

China (Anhui, Chongqing, Guangxi, Guizhou, Hubei, Hunan, Jiangxi, Sichuan and Yunnan).

### 5. Nekemias gongshanensis

Taxon classificationAnimaliaVitalesVitaceae

(C.L. Li) J. Wen & Z.L. Nie comb. nov.

urn:lsid:ipni.org:names:77142318-1

Ampelopsis gongshanensis C.L. Li, Chinese J. Appl. Environ. Biol. 2(1): 48. 1996. Basionym

#### Distribution.

China (Yunnan).

### 6. Nekemias grossedentata

Taxon classificationAnimaliaVitalesVitaceae

(Hand.-Mazz.) J. Wen & Z.L. Nie comb. nov.

urn:lsid:ipni.org:names:77142325-1

Ampelopsis cantoniensis var. grossedentata Hand.-Mazz., Sitzungsber. Kaiserl. Akad. Wiss., Math.-Naturwiss. Cl., Abt. 1, 59: 105. 1877. Basionym
Ampelopsis grossedentata (Hand.-Mazz.) W.T. Wang, Acta Phytotax. Sin. 17(3): 79. 1979.

#### Distribution.

China (Fujian, Guangdong, Guangxi, Guizhou, Hubei, Hunan, Jiangxi and Yunnan).

### 7. Nekemias hypoglauca

Taxon classificationAnimaliaVitalesVitaceae

(Hance) J. Wen & Z.L. Nie comb. nov.

urn:lsid:ipni.org:names:77142319-1

Hedera hypoglauca Hance, Ann. Bot. Syst. 2: 724. 1852. Basionym
Ampelopsis hypoglauca (Hance) C.L. Li, Chinese J. Appl. Environ. Biol. 2(1): 48. 1996.

#### Distribution.

China (Fujian, Guangdong, Hong Kong and Jiangxi).

### 8. Nekemias megalophylla

Taxon classificationAnimaliaVitalesVitaceae

(Diels & Gilg) J. Wen & Z.L. Nie comb. nov.

urn:lsid:ipni.org:names:77142320-1

Ampelopsis megalophylla Diels & Gilg, Bot. Jahrb. Syst. 29: 466. 1900. Basionym

### 8a. Nekemias megalophylla (Diels & Gilg) J. Wen & Z.L. Nie var. megalophylla

Taxon classificationAnimaliaVitalesVitaceae

Vitaeda megalophylla (Diels & Gilg) Börner, Abh. Nat. Ver. Bremen 21: 280. 1913.

#### Distribution.

China (Chongqing, Gansu, Guizhou, Hubei, Shaanxi, Sichuan and Yunnan).

### 8b. Nekemias megalophylla var. jiangxiensis

Taxon classificationAnimaliaVitalesVitaceae

(W.T. Wang) J. Wen & Z.L. Nie comb. nov.

urn:lsid:ipni.org:names:77142321-1

Ampelopsis jiangxiensis W.T. Wang, Bull. Bot. Res. North-East. Forest. Inst. 1(1–2): 170. 1981. Basionym
Ampelopsis megalophylla var. jiangxiensis (W.T. Wang) C.L. Li, Chinese J. Appl. Environ. Biol. 2(1): 48. 1996.

#### Distribution.

China (Jiangxi).

### 9. Nekemias rubifolia

Taxon classificationAnimaliaVitalesVitaceae

(Wall.) J. Wen & Z.L. Nie comb. nov.

urn:lsid:ipni.org:names:77142322-1

Vitis rubifolia Wall., Fl. Ind., ed. Carey & Wall., 2: 480. 1824. Basionym
Ampelopsis rubifolia (Wall.) Planch., Monogr. Phan. 5: 463. 1887.
Ampelopsis megalophylla var. puberula W.T. Wang, Acta Phytotax. Sin. 17(3): 79, 90. 1979.

#### Distribution.

China (Guangxi, Guizhou, Hunan, Jiangxi and Sichuan), and India.

### Taxonomic key to species of *Nekemias*

**Table:** 

1	Leaves abaxially strongly glaucous	**2**
1	Leaves green on both surfaces	**3**
2	Lower leaves pinnately compound, leaflet blades 7–15 × 3–7 cm	***Nekemias chaffanjonii***
2	Lower leaves bipinnately compound, leaflet blades 2.5–6 × 1–3.5 cm	***Nekemias hypoglauca***
3	Leaves pinnately compound	**4**
3	Leaves bipinnately, ternately bipinnately to tripinnately compound	**5**
4	Leaflets 3.5–14 × 2–6.5 cm, margin 5–15-toothed, abaxially densely ferruginous pilose; berries 8–15 mm in diameter	***Nekemias rubifolia***
4	Leaflets 3–6 × 0.5–3 cm, margin entire or with 1 to several inconspicuous teeth, midvein abaxially sparsely pilose; berries 5–7 mm in diameter	***Nekemias gongshanensis***
5	Tendril trifurcate; leaflets 4–12 × 2–6 cm	***Nekemias megalophylla***
5	Tendril bifurcate; leaflets 1–5 × 0.5–2.5 cm	**6**
6	Leaflet margin with 2–4 large coarse teeth; from North America or the Caribbean	***Nekemias arborea***
6	Leaflet margin serrate with 5–15 teeth on each side; from Asia	**7**
7	Leaflet margin coarsely serrate, central leaflet ovate-elliptical	***Nekemias grossedentata***
7	Leaflet margin ± undulate, central leaflet obovate or ovate	**8**
8	Leaves and inflorescences pilose to glabrescent	***Nekemias cantoniensis***
8	Leaves and inflorescences pubescent to densely so	***Nekemias celebica***

## Supplementary Material

XML Treatment for Nekemias

XML Treatment for Nekemias arborea

XML Treatment for Nekemias cantoniensis

XML Treatment for Nekemias celebica

XML Treatment for Nekemias chaffanjonii

XML Treatment for Nekemias gongshanensis

XML Treatment for Nekemias grossedentata

XML Treatment for Nekemias hypoglauca

XML Treatment for Nekemias megalophylla

XML Treatment for Nekemias megalophylla (Diels & Gilg) J. Wen & Z.L. Nie var. megalophylla

XML Treatment for Nekemias megalophylla var. jiangxiensis

XML Treatment for Nekemias rubifolia
